# Investigating the removal of some pharmaceutical compounds in hospital wastewater treatment plants operating in Saudi Arabia

**DOI:** 10.1007/s11356-016-6389-7

**Published:** 2016-03-21

**Authors:** Hamed Al Qarni, Philip Collier, Juliette O’Keeffe, Joseph Akunna

**Affiliations:** Urban Water Technology Centre, School of Science, Engineering and Technology, University of Abertay Dundee, Bell Street, Dundee, DD1 1HG Scotland UK

**Keywords:** Pharmaceutical compounds, Wastewater, Temperature, Removal rates, Saudi Arabia, Desert climate, Activated sludge

## Abstract

The concentrations of 12 pharmaceutical compounds (atenolol, erythromycin, cyclophosphamide, paracetamol, bezafibrate, carbamazepine, ciprofloxacin, caffeine, clarithromycin, lidocaine, sulfamethoxazole and N-acetylsulfamethoxazol (NACS)) were investigated in the influents and effluents of two hospital wastewater treatment plants (HWWTPs) in Saudi Arabia. The majority of the target analytes were detected in the influent samples apart from bezafibrate, cyclophosphamide, and erythromycin. Caffeine and paracetamol were detected in the influent at particularly high concentrations up to 75 and 12 ug/L, respectively. High removal efficiencies of the pharmaceutical compounds were observed in both HWWTPs, with greater than 90 % removal on average. Paracetamol, sulfamethoxazole, NACS, ciprofloxacin, and caffeine were eliminated by between >95 and >99 % on average. Atenolol, carbamazepine, and clarithromycin were eliminated by >86 % on average. Of particular interest were the high removal efficiencies of carbamazepine and antibiotics that were achieved by the HWWTPs; these compounds have been reported to be relatively recalcitrant to biological treatment and are generally only partially removed. Elevated temperatures and high levels of sunlight were considered to be the main factors that enhanced the removal of these compounds.

## Introduction

Pharmaceutical compounds include a wide range of chemicals with different structures, functions, behaviors, and activities and are used to enhance human health in the medical field. After their excretion by patients, these compounds and their metabolites can contaminate surface water, ground water, and drinking water (Kolpin et al. 2002; Monteiro and Boxall 2010; Li et al. 2013; Schaider et al. 2014). The main sources of these compounds and their metabolites in aquatic environments are wastewater treatment plants (but can also include sources such as manufacturing wastes and veterinary sources) (Vieno et al. 2007; Phillips et al. 2010; Vulliet et al. 2011; Tewari et al. 2013). Previous studies have detected high concentrations of some pharmaceuticals in hospital wastewaters (Ohlsen et al. 2003; Kosma et al. 2010; Kovalova et al. 2012). Hospital wastewater is, in most cases, connected directly to urban sewer systems without pretreatment, so municipal wastewaters are usually cotreated with hospital wastewater in municipal wastewater treatment plants (MWWTPs) (Alder et al. 2006); however, municipal systems are not usually designed to remove medical or pharmaceutical wastes.

Various methods for the removal of pharmaceuticals from wastewater have been studied such as conventional activated sludge (AS) (Oppenheimer et al. 2007; Nielsen et al. 2013) membrane bioreactors (MBRs) (Clara et al. 2005; Radjenović et al. 2009; Kim et al. 2014; Verlicchi et al. 2010; Kovalova et al. 2012), and moving bed biofilm reactors (MBBRs) (Escola Casas et al. 2015) as shown in Table 1 with regard to compounds analyzed in this study. Currently, the AS process is the most common treatment process employed at wastewater treatment plants; previous studies have indicated a significant variation in the removal of pharmaceutical compounds during treatment using the AS process, ranging from complete removal (e.g., paracetamol and ibuprofen) to poor removal (e.g., carbamazepine). Differences between removal rates from various processes have also been studied. No significant differences in the removal efficiency of certain compounds (e.g., ibuprofen, triclosan, and caffeine) were found between the MBR and conventional AS processes by Oppenheimer et al. (2007). Pilot and laboratory-scale experiments by Nielsen et al. (2013) found that removal of many active pharmaceutical ingredients could be effectively achieved using MBR plus ozone, ozone + hydrogen peroxide or powdered activated carbon (PAC), with MBR + PAC being the most efficient technology. MBBRs were found to be a potentially promising solution for treatment of hospital wastewater, with high elimination rates (>80 %) observed for some compounds (ibuprofen, propranolol, acetyl-sulfadiazine) in batch experiments, however, with low elimination rates (<20 %) observed for others (sulfamethoxazole, venlafaxine, iopromide, tramadol, and diatrizoic) (Escola Casas et al. 2015).

**Table 1:** Comparison of removal efficiencies of pharmaceutical compounds in various wastewater treatment processes for hospital wastewater (HWWTP) and municipal wastewater (MWWTP)

Compound	Treatment plant type	Removal (%)	Reference
Paracetamol	HWWTP AS + disinfection (GR)	75	Kosma et al. (2010)
	MWWTP AS + sand filter (GR)	95.6	Kosma et al. (2010)
	MWWTP AS + trickling filter (UK)	94	Kasprzyk-Hordern et al. (2009)
	MWWTP (+ industrial) AS (UK)	>99	Kasprzyk-Hordern et al. (2009)
	HWWTP MBR (pilot scale) (CH)	>99	Kovalova et al. (2012)
	HWWTP MBR (pilot scale) (ceramic UF) (DK)	>99	Nielsen et al. (2013)
Carbamazepine	HWWTP AS + disinfection (GR)	30	Kosma et al. (2010)
	MWWTP AS + sand filter (GR)	NR	Kosma et al. (2010)
	MWWTP (+ industrial) AS (ES)	NR	Radjenović et al. (2009)
	MWWTP (+ industrial) MBR^c^ (pilot scale) (ES)	NR	Radjenović et al. (2009)
	MWWTP (+ industrial) MBR^d^ (pilot scale) (ES)	NR	Radjenović et al. (2009)
	HWWTP MBBR (pilot scale) (DK)	10	Escola Casas et al. (2015)
	MWWTP AS + trickling filter (UK*)*	NR	Kasprzyk-Hordern et al. (2009)
	MWWTP (+ industrial) AS (UK)	13	Kasprzyk-Hordern et al. (2009)
	HWWTP MBR (pilot scale) (CH)	−6 ± 12	Kovalova et al. (2012)
	HWWTP MBR (pilot scale) (ceramic UF) (DK)	1	Nielsen et al. (2013)
	MWWTP MBR (hollow fiber membrane)	28	Kim et al. (2014)
Atenolol	MWWTP (+ industrial) AS (ES)	61.2 ± 18.6	Radjenović et al. (2009)
	MWWTP (+ industrial) MBR^c^ (pilot scale) (ES)	76.7 ± 12.6	Radjenović et al. (2009)
	MWWTP (+ industrial) MBR^d^ (pilot scale) (ES)	69.5 ± 12.5	Radjenović et al. (2009)
	HWWTP MBBR (pilot scale) (DK)	40	Escola Casas et al. (2015)
	MWWTP AS + trickling filter (UK)	78	Kasprzyk-Hordern et al. (2009)
	MWWTP (+ industrial) AS (UK)	85	Kasprzyk-Hordern et al. (2009)
	HWWTP MBR (pilot scale) (CH)	99 ± 1	Kovalova et al. (2012)
	HWWTP MBR (pilot scale) (ceramic UF) (DK)	70	Nielsen et al. (2013)
	MWWTP MBR (hollow fiber membrane) (CAN)	77	Kim et al. (2014)
Bezafibrate	MWWTP (+ industrial) AS (ES)	80.8 ± 20.9	Radjenović et al. (2009)
	MWWTP (+ industrial) MBR^c^ (pilot scale) (ES)	90.3 ± 10.1	Radjenović et al. (2009)
	MWWTP (+ industrial) MBR^d^ (pilot scale) (ES)	88.2 ± 15.3	Radjenović et al. (2009)
	MWWTP AS + trickling filter (UK)	45	Kasprzyk-Hordern et al. (2009)
	MWWTP (+ industrial) AS (UK)	71	Kasprzyk-Hordern et al. (2009)
	HWWTP MBR (pilot scale) (CH)	>91	Kovalova et al. (2012)
Lidocaine	HWWTP MBR (pilot scale) (CH)	56 ± 13	Kovalova et al. (2012)
Ciprofloxacin	HWWTP MBR (pilot scale) (CH)	51 ± 13	Kovalova et al. (2012)
	HWWTP MBR (pilot scale) (ceramic UF) (DK)	36	Nielsen et al. (2013)
	MWWTP MBR (hollow fiber membrane) (CAN)	89	Kim et al. (2014)
Clarithromycin	HWWTP MBR (pilot scale) (CH)	50 ± 12	Kovalova et al. (2012)
	MWWTP AS + UV (TW)	NR	Lin et al. (2010)
	MWWTP AS + chlorination (TW)	NR	Lin et al. (2010)
	MWWTP AS + chlorination (TW)	10	Lin et al. (2010)
	MWWTP AS + sand filer (TW)	NR	Lin et al. (2010)
	MWWTP trickling filter + chlorination (TW)	99	Lin et al. (2010)
	MWWTP AS + chlorination (TW)	NR	Lin et al. (2010)
	HWWTP MBR (pilot scale) (ceramic UF) (DK)	64	Nielsen et al. (2013)
	MWWTP MBR (hollow fiber membrane) (CAN)	NR	Kim et al. (2014)
Sulfamethoxazole	MWWTP (+ industrial) AS (ES)	73.8 ± 12.7	Radjenović et al. (2009)
	MWWTP (+ industrial) MBR^c^ (pilot scale) (ES)	80.8 ± 12.2	Radjenović et al. (2009)
	MWWTP (+ industrial) MBR^d^ (pilot scale) (ES)	78.3 ± 13.9	Radjenović et al. (2009)
	HWWTP MBBR (pilot scale) (DK)	NR	Escola Casas et al. (2015)
	MWWTP AS + trickling filter (UK)	66	Kasprzyk-Hordern et al. (2009)
	MWWTP (+ industrial) AS (UK)	83	Kasprzyk-Hordern et al. (2009)
	HWWTP MBR (pilot scale) (CH)	7 ± 57	Kovalova et al. (2012)
	MWWTP AS + UV (TW)	42	Lin et al. (2010)
	MWWTP AS + chlorination (TW)	20	Lin et al. (2010)
	MWWTP AS + chlorination (TW)	59	Lin et al. (2010)
	MWWTP AS + sand filter (TW)	88	Lin et al. (2010)
	MWWTP trickling filter + chlorination (TW)	45	Lin et al. (2010)
	MWWTP AS + chlorination (TW)	26	Lin et al. (2010)
	HWWTP MBR (pilot scale) (ceramic UF) (DK)	97	Nielsen et al. (2013)
	MWWTP MBR (hollow fiber membrane) (CAN)	66	Kim et al. (2014)
Erythromycin	MWWTP (+ industrial) AS (ES)	35.4 ± 50.5	Radjenović et al. (2009)
	MWWTP (+ industrial) MBR^c^ (pilot scale) (ES)	43.0 ± 51.5	Radjenović et al. (2009)
	MWWTP (+ industrial) MBR^d^ (pilot scale) (ES)	25.2 ± 108.9	Radjenović et al. (2009)
	HWWTP MBBR (pilot scale) (DK)	<20	Escola Casas et al. (2015)
	MWWTP AS + trickling filter (UK^a^)	14	Kasprzyk-Hordern et al. (2009)
	MWWTP (+ industrial) AS (UK^a^)	72	Kasprzyk-Hordern et al. (2009)
	HWWTP MBR (pilot scale) (CH^b^)	<60	Kovalova et al. (2012)
	MWWTP AS + UV (TW^a^)	NR	Lin et al. (2010)
	MWWTP AS + chlorination (TW^a^)	NR	Lin et al. (2010)
	MWWTP AS + chlorination (TW^a^)	77	Lin et al. (2010)
	MWWTP AS + sand filter (TW^a^)	NR	Lin et al. (2010)
	MWWTP trickling filter + chlorination (TW^a^)	56	Lin et al. (2010)
	MWWTP AS + chlorination (TW^a^)	NR	Lin et al. (2010)
	HWWTP MBR (pilot scale) (ceramic UF) (DK)	37	Nielsen et al. (2013)
	MWWTP MBR (hollow fiber membrane) (CAN^a^)	12	Kim et al. (2014)
Cyclophosphamide	HWWTP MBR (pilot scale) (CH)	<20	Kovalova et al. (2012)
	HWWTP MBR (pilot scale) (ceramic UF) (DK)	12	Nielsen et al. (2013)
Caffeine	HWWTP AS + disinfection (GR)	75	Kosma et al. (2010)
	MWWTP AS + sand filter (GR)	89	Kosma et al. (2010)
	MWWTP AS + UV (TW)	99	Lin et al. (2010)
	MWWTP AS + chlorination (TW)	>99	Lin et al. (2010)
	MWWTP AS + chlorination (TW)	97	Lin et al. (2010)
	MWWTP AS + sand filter (TW)	99	Lin et al. (2010)
	MWWTP trickling filter + chlorination (TW)	96	Lin et al. (2010)
	MWWTP AS + chlorination (TW)	>99	Lin et al. (2010)
	MWWTP MBR (hollow fiber membrane) (CAN)	100	Kim et al. (2014)

*AS* activated sludge, *MBR* membrane bioreactor, *MBBR* moving bed biofilm reactor, *UF* ultrafiltration, *NR* no removal, *CAN* Canada, *CH* Switzerland, *DK* Denmark, *ES* Spain, *GR* Greece, *TW* Taiwan, *UK* United Kingdom

^a^Erythromycin·H_2_O

^b^Erythromycin + Eryt·H_2_O (30–100 %)

^c^Flat sheet microfiltration

^d^Hollow fiber UF

Some studies have also investigated the biodegradation efficiency of some pharmaceutical compounds under anaerobic processes (Carballa et al. 2007; Musson et al. 2010). The reported biodegradation efficiency has varied from no elimination to high elimination. For example, Carballa et al. (2007) observed significant elimination rates for some antibiotics (sulfamethoxazole) and natural estrogens, while there was no elimination of carbamazepine. Musson et al. (2010) investigated the fate of six pharmaceutical compounds (17α-ethynylestradiol, acetaminophen, acetylsalicylic acid, ibuprofen, metoprolol tartrate, and progesterone) during anaerobic digestion and only found a significant biodegradation potential for acetylsalicylic acid.

The level of removal efficiency by biological treatments depends on the physicochemical properties of the compounds, the type of wastewater treatment technology, the hydraulic retention time (HRT), the solids retention time (SRT), and the climatic conditions (e.g., dilution, rainfall, temperature, and level of sunlight) (Kasprzyk-Hordern et al. 2009; McAdam et al. 2010; Sahar et al. 2011). The variation mainly occurs because these parameters, and other physicochemical properties of compounds, affect microbial activity and growth, thereby resulting in a change in effluent quality (Pollice et al. 2002; Fernandez-Fontaina et al. 2012; Arévalo et al. 2014; Chen et al. 2014).

Temperature conditions in biological wastewater treatment processes can significantly affect microbial activity and growth (LaPara et al. 2000; Vieno et al. 2005; Massmann et al. 2006; Calderón et al. 2012). Previous studies in this field have only investigated the removal efficiency of pharmaceutical compounds in AS in response to seasonal variations in temperature in Europe and North America (average <20 °C). Relatively low removal efficiencies have been observed, especially during the winter period (Heberer 2002; Kolpin et al. 2002; Metcalfe et al. 2003; Miao et al. 2005). However, temperature stability in the biological wastewater treatment processes may be an important factor in micropollutant removal, particularly in arid and semiarid areas, where the average annual temperature is >25 °C. In arid and semiarid areas, such as Saudi Arabia, the diurnal temperature during summer can range between 30 and 55 °C, with an annual average of above 30 °C (Aksakal and Rehman 1999; Qadir et al. 2010; Almazroui et al. 2014). This will consequentially result in higher temperatures in wastewaters compared to both winter and summer conditions in temperate countries. In addition to temperature, scarcity of available rainfall and intense sunlight can affect removal efficiency (Hai et al. 2011). This study aims to investigate the occurrence and fate of selected pharmaceutical compounds at onsite hospital wastewater treatment plants (HWWTPs) in Riyadh (Saudi Arabia) operating under high ambient temperature conditions.

## Materials and methods

### Treatment plant selection

Two HWWTPs were selected in central Saudi Arabia, both of which were located in Riyadh. The choice of these locations was based on the following factors: (i) the HWWTPs performed onsite treatment; and (ii) the HWWTPs at both hospitals employed AS processes. As far as was known, all other HWWTPs in Riyadh (in fact, the entire country) employed AS processes; therefore, the sites selected in this study should generally be representative of HWWTPs in Saudi Arabia. The known operational parameters at the two HWWTPs are described in Table 2. For the purposes of this study, the sample collection locations are referred to as sites HWWTP1 and HWWTP2. Due to limited access to data, some information on the operational processes at the HWWTPs was not available (e.g., the HRT and SRT, among other operational parameters that are commonly measured). The wastewater at each hospital consists of a similar combination of units including outpatients, inpatients, medical units, restaurants, and laundry, with the only difference being the absence of a urology unit at HWWTP2.

**Table 2:** Overview of the operational parameters at the two hospital wastewater treatment plants studied

Parameters	HWWTP1		HWWTP2	
Number of beds	300		215	
Annual wastewater volume (m^3^)	330,000		227,000	
Process technology	Aerobic		Aerobic	
Ambient (°C)	28 ± 7		28 ± 7	
pH	7.0–7.5		6.8–7.5	
	Influent	Effluent	Influent	Effluent
COD (mg/L)	376	64	336	27
NH_4_^+^ (mg/L)	22.3	0.3	19.0	0
NO_2_^−^ (mg/L)	0.3	0.6	0.3	0.3
NO_3_^−^ (mg/L)	0.2	0.7	0.4	2.4

### Sampling

**Fig. 1:**
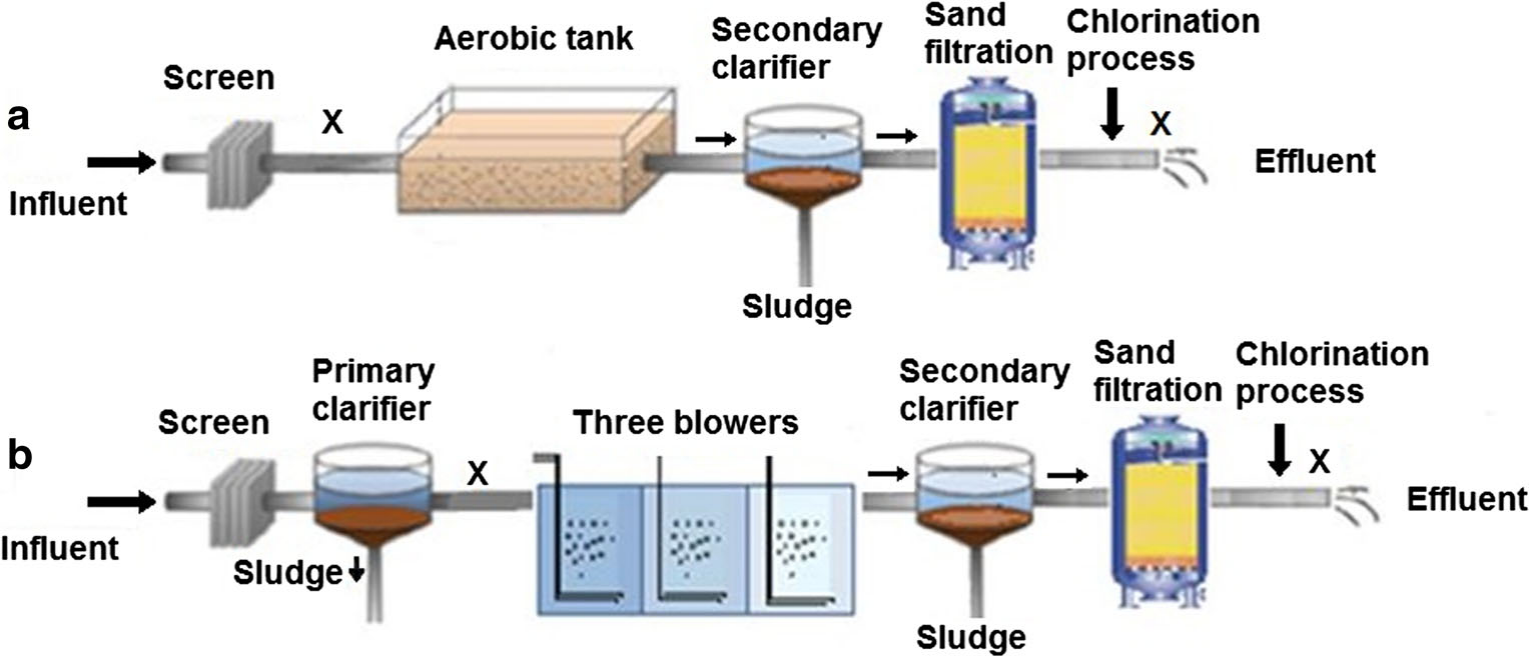
Schematic of the treatment processes employed in the **a** hospital wastewater treatment plant 1 and **b** hospital wastewater treatment plant 2; (X = sampling point)

Figure 1 summarizes the treatment processes used at each plant. Samples were collected from the influent (before the secondary treatment) and effluent (after the tertiary treatment) of each HWWTP. Sampling was carried out twice weekly for 4 weeks in April 2014. One sample of influent and one sample of effluent were collected on each sampling day. Samples were collected in the middle of the day (between 11:00 and 14:00) when the air temperature was at its highest (30–35 °C). The samples were collected by grab sampling in 1000-mL sterile plastic bottles (Saudi Water, Saudi Arabia) and transferred to the laboratory in a cool box, and then frozen at −20 °C. All the samples collected from each respective sampling point (influent and effluent) (8 × 1000 ml each) were mixed together to provide a composite sample for the sampling period. Three aliquots of 1000 mL of the mixed samples for each site were then taken for analysis. This allowed an assessment of the levels of pharmaceutical compounds present in the influents and effluents of the HWWTPs in the middle of the day, which likely represented the highest daily water temperatures and the maximum loads received by the HWWTPs (Ort et al. 2010).

### Analysis of pharmaceutical compounds

The pharmaceutical compounds investigated in this study were representative of the wide range of pharmaceutical compounds commonly present in municipal wastewaters (Yu et al. 2006; Lin et al. 2010; Helwig et al. 2013). These compounds included antibiotics, analgesics, β-blockers, anesthetics, anticonvulsants, cytostatic antineoplastics, and lipid regulators. Caffeine concentrations were also analyzed. Sample analysis was carried out by an external laboratory (School of Engineering and Built Environment, Glasgow Caledonian University, United Kingdom).

#### Sample preparation

The pharmaceutical compounds were extracted using solid phase extraction (SPE). The triplicate subsamples (1000 mL) from each of the sampling points were filtered sequentially through 100-, 1.6-, and 0.7-μm glass microfiber filters (Whatman, UK), and then through a 0.45-μm cellulose nitrate membrane sterile filter (Whatman, UK). Each filtered sample was adjusted to pH 2.0 (±0.1) with the addition of 0.5 M hydrochloric acid. Prior to extraction, the Strata-X (1-g/20-mL, 33 μ polymeric reversed-phase) cartridges (Phenomenex, UK) were preconditioned with 2 mL of methanol and 2 mL of distilled water. Subsequently, the samples were loaded at a flow rate of 10 mL/min using an SPE vacuum manifold with 12 accessories (Macherey-Nagel, Germany). The cartridges were dried under a vacuum. After extraction, the cartridges were washed with 3 × 2-mL water (18 MΩ cm) and then eluted with 4 × 2-mL CH_3_CN/MeOH containing 0.1 % formic acid. The samples were then dried under nitrogen gas. The dried samples were reconstituted into CH_3_CN/H_2_O (30/70). The samples were then diluted further (1:8) by 100-μL aliquot and adding 700 μL of CH_3_CN/H_2_O. The dilution of samples helped to minimize matrix effects; however, this was not completely eliminated. Acetonitrile used was Optima grade (Fisher). Deuterated internal standards were added to afford a concentration of 5 μg/L prior to liquid chromatography/tandem mass spectrometry (LC–MS/MS) analysis. Deuterated internal standards used were atenolol (d7), carbamazepine (d8), lidocaine (d10), paracetamol (d3), and caffeine (d3), purchased from CDN isotopes. Pharmaceutical standards used were Sigma-Aldrich HPLC standard grade.

#### LC–MS/MS analysis

Each composite sample was analyzed in triplicate. Chromatographic separation of the analytes was performed using LC–MS/MS (Thermo Scientific Q Exactive Quadrupole–Orbitrap Mass Spectrometer) equipped with a column: Waters Xselect HSS T3. 2.1 × 150 mm (Water, UK). The MS positive ion mode and experiment type are listed in Table 3 along with linear range, retention times, and limits of quantification (LOQs). The samples were bracketed by calibration line and quality control standards (calibration line criteria of ±20 % linear calibration with a weight of 1/x^2^). Conditions included electrospray ionization, positive ion mode, full scan range of 100–1000, mass resolution of 17,500, targeted MS2 mass resolution of 17,500, spray voltage of 3.5 kV, capillary temperature of 300 °C, and auxiliary gas heater of 300 °C. Some compounds were analyzed in MS2, while those that fragmented less well were analyzed in full scan mode.

**Table 3:** Mass spectrometry: positive ion mode, range, and limit of quantification (LOQ)

Analyte	MS ion/transition^a^	Retention time (min)	MS experiment	Linear range (ng/ml)	LOQ (ng/ml)
Atenolol	267.1703	6.38	Full scan	0.1–500	5
Bezafibrate	362.11–316.1089	19.63	Targeted MS2	0.1–500	0.1
Carbamazepine	237.10–194.0964	17.72	Targeted MS2	0.25–500	0.25
Caffeine	195.0877	7.20	Full scan	2.5–500	2.5
Ciprofloxacin	332.14–288.1505	7.43	Targeted MS2	0.25–500	2.5
Clarithromycin	748.48–158.1176	19.08	Targeted MS2	0.1–250	0.5
Cyclophosphamide	261.03–140.0029	10.68	Targeted MS2	0.5–500	1
Erythromycin	734.47–158.1176	14.99	Targeted MS2	0.5–500	0.5
Lidocaine	235.1805	7.88	Full scan	1–500	1
NACS	296.07–134.0602	10.69	Targeted MS2	2.5–500	5
Paracetamol	152.0706	6.80	Full scan	0.5–500	0.5
Sulfamethoxazole	254.06–108.0445	10.59	Targeted MS2	1–500	1

^a^The underlined m/z being used for quantification

## Results and discussion

### Occurrence of pharmaceutical compounds in hospital wastewater

Out of the 12 compounds analyzed, 9 pharmaceutical compounds were detected, including 3 antibiotic compounds (ciprofloxacin, clarithromycin, sulfamethoxazole) and the antibiotic metabolite, NACS, one analgesic (paracetamol), one stimulant (caffeine), one β-blocker (atenolol), one anesthetic (lidocaine), and one anticonvulsant (carbamazepine). The other compounds tested for, including bezafibrate, erythromycin, and cyclophosphamide were not detected in either the influents or effluents of the HWWTPs. Mean concentrations are presented in Table 4. Removal efficiencies were calculated as difference between mean concentrations in influent and effluent.

**Table 4:** Concentrations of pharmaceutical compounds in hospital wastewater treatment plants in Saudi Arabia (ng/L)^a^

Class	Compound	LOQ^b^	HWWTP1	HWWTP1	HWWTP2	HWWTP2
			Inf.	Eff.	Inf.	Eff.
Analgesics	Paracetamol	0.5	12400 ± 340	73 ± 11	12300 ± 180	157 ± 20
Antidepressants	Carbamazepine	0.25	151 ± 13	41 ± 1	73 ± 14	n/d
β-blockers	Atenolol	5.0	730 ± 82	46 ± 2	329 ± 28	55 ± 4
Lipid regulators	Bezafibrate	0.1	n/d	n/d	n/d	n/d
Anesthetics	Lidocaine	1.0	158 ± 12	114 ± 4	129 ± 6	<LOQ
Antibiotics	Ciprofloxacin	2.5	5600 ± 660	n/d	2180 ± 250	n/d
	Clarithromycin	0.5	83 ± 72	22 ± 9	38	n/d
	Sulfamethoxazole	1.0	30 ± 7	n/d	132 ± 5	n/d
	Erythromycin	0.5	n/d	n/d	n/d	n/d
*Metabolite of sulfamethoxazole*	NACS	5.0	1200 ± 55	59 ± 14	506 ± 21	n/d
Cytostatic	Cyclophosphamide	1.0	n/d	n/d	n/d	n/d
Others	Caffeine	2.5	74,800 ± 15,500,502	n/d	27,500 ± 2000	n/d

*n/d* not detected

^a^Each sample was analyzed in triplicate. Results are reported as mean ± standard deviation (*n* = 3)

^b^Limit of quantitation (substances detected but not quantifiable); *n/d* = not detected

Caffeine and paracetamol were detected in all of the influent samples and were present at the highest concentrations of all the compounds analyzed, up to 7479 and 12,390 ng/L, respectively. Especially high concentrations of caffeine (>90 μg/L) were found at the HWWTP1 influent. Caffeine has been detected in MWWTPs in China at concentrations at 3.4–6.6 μg/L (Sui et al. 2010), in Switzerland at 7–73 μg/L (Buerge et al. 2003), and in Spain at concentrations up to 89 μg/L (Martín et al. 2012). Alidina et al. (2014) reported high concentrations of caffeine (64–16,500 ng/L) in the effluent of six Saudi MWWTPs (but no data were available for influent concentrations in their study). The high concentrations of caffeine observed in this study may be related to its administration in combination with other medication in order to enhance the effects of certain analgesics in cough, cold, and headache medicines (Lin et al. 2010; Weigel et al. 2002). It is also used as a cardiac, cerebral, and respiratory stimulant and as a diuretic (Buerge et al. 2003). Both caffeine and paracetamol were almost completely removed during the treatment process in both of the HWWTPs. Negligible concentrations of paracetamol were detected in the both HWWTP effluents, while the removal efficiencies for caffeine were near 100 %.

As expected, the hospital influents were found to contain high levels of antibiotics and three of the antibiotic compounds examined (ciprofloxacin, clarithromycin, sulfamethoxazole) and the sulfamethoxazole metabolite, NACS, were detected at concentrations ranging from 30 to 5611 ng/L in the influents of the HWWTPs. The high concentrations of the antibiotics in the raw hospital wastewater are likely to be present due to the high levels of antibiotic consumption in hospitals (Kümmerer 2009). Ciprofloxacin was present in both influents at the highest concentrations of the antibiotic compounds tested, followed by NACS at concentrations up to 5611 and 1234 ng/L, respectively. Relatively low concentrations (<160 ng/L) of sulfamethoxazole and clarithromycin were detected in the raw hospital wastewater. Previous studies have also found high concentrations of sulfamethoxazole (730 ng/L) in Saudi effluents from MWWTPs (Alidina et al. 2014). In this study, the concentrations of all the antibiotics in the effluents of the HWWTPs were found to be negligible and lower than their respective predicted no effect concentrations (PNECs) reported in the literature of 9.38 × 10^5^ ng/L for ciprofloxacin, 70 ng/L for clarithromycin, and 27 ng/L for sulfamethoxazole (Verlicchi et al. 2012).

Carbamazepine, atenolol, and lidocaine were consistently detected in the influent samples of the HWWTPs at relatively low concentrations (<1 μg/L). Previously, atenolol was detected at low concentrations (1–4 ng/L) in the influent of a Saudi MWWTP (Shraim et al. 2012). These drugs have been considered relatively recalcitrant to biological treatment and are generally only partially removed in wastewater treatment systems (Zhang et al. 2008; Paxeus 2004; Rúa-Gómez and Püttmann 2012). In this study, negligible concentrations of these drugs were detected in the effluents of the HWWTPs. These concentrations are lower than their respective PNECs of 1.38 × 10^4^ ng/L for carbamazepine and 3.0 × 10^4^ ng/L for atenolol (Verlicchi et al. 2012) and the PNEC of 1.06 × 10^5^ ng/L for lidocaine as reported by AstraZeneca (2013). The concentrations of atenolol in effluent fall within the lower range of concentrations previously report for effluents of various MWWTPs in Saudi Arabia (15–2550 ng/L); however, carbamazepine in effluent was lower than levels previously reported (57–1200 ng/L) (Alidina et al. 2014).

### Potential influence of the operational parameters on removal efficiencies

**Fig. 2:**
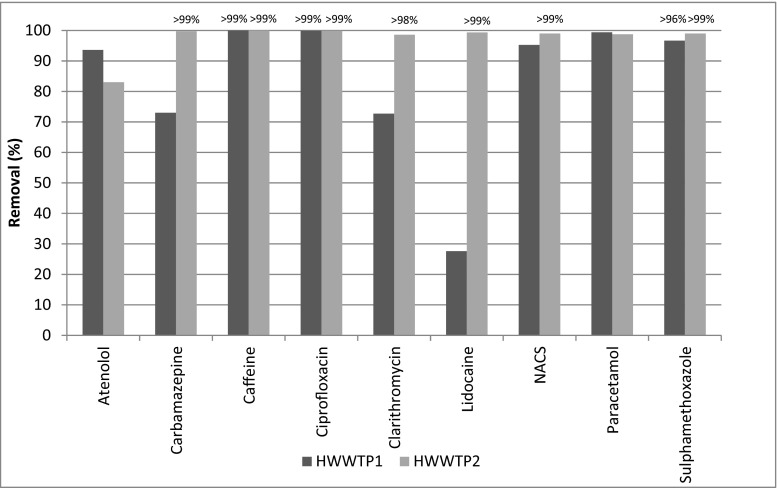
Removal efficiencies of pharmaceutical compounds at HWWTP1 and HWWPT2 in Saudi Arabia. Results shown are mean removal (*n* = 3)

The removal efficiencies of pharmaceutical compounds from the hospital wastewater samples show that, on average, the highest decreases in effluent concentrations were observed for ciprofloxacin, caffeine, sulfamethoxazole, paracetamol, and NACS (> 95 %), followed by atenolol, carbamazepine, and clarithromycin (>85 %). The average removal efficiency of lidocaine was greater than 65 % at the two HWWTPs (Fig. 2).

#### Paracetamol and caffeine

Caffeine and paracetamol were both almost completely removed during treatment in each of the HWWTP. The average removal efficiency of paracetamol was >98 %, while the caffeine was nearly completely (>99 %) removed by both HWWTPs. Similar results were obtained for the removal of paracetamol (up to 99 %) in AS processes by Kasprzyk-Hordern et al. (2009) and Verlicchi et al. (2013), under temperate climate conditions. Thus, paracetamol removal at WWTPs seems possible using conventional ASP under wider climate conditions (in both cold and warm weather). With regard to the removal of caffeine, Lin et al. (2010) reported similar caffeine removal efficiencies (99 %) in six Taiwanese WWTPs. In contrast to these results, Buerge et al. (2003) found that the removal efficiencies of caffeine varied (81–99 %) at 13 Swiss MWWTPs. The authors indicated that the MWWTPs that were less efficient at removing caffeine (81 %) employed lower adaptation times for the microorganisms in the AS system (<5 days versus >5 days). Bacterial adaptation in AS systems is enhanced through longer SRTs and higher temperatures (Batt et al. 2006). Therefore, in this study, the effective biodegradation of both compounds may have resulted from the high ambient temperatures and/or the possibility that both plants may have been operating with HRTs and SRTs that encouraged greater microbial adaptation.

#### Antibiotics

The mean removal efficiencies of ciprofloxacin, clarithromycin, sulfamethoxazole, and NACS were >99, 86, >98, and 97 %, respectively. The HWWTP2 achieved almost complete removal of the antibiotics. Lin et al. (2009) previously studied sulfamethoxazole and clarithromycin and reported that the removal efficiencies of six different Taiwan WWTPs were greater than 50 and 20 %, respectively. Carballa et al. (2004) also found a lower removal efficiency (<60 %) of sulfamethoxazole in an AS plant located in temperate climate conditions (Galicia, Spain). A seasonal variation in the removal of sulfamethoxazole was observed, with a higher removal rate in summer (71 %) than in winter (17 %), in Italy (Castiglioni et al. 2006). These results, and others listed in Table 1, are all much lower than the results observed in this study. Other studies observed the deconjugation of its metabolite (NACS), and thus higher concentrations of sulfamethoxazole in the effluent compared to the influent (Ashton et al. 2004; Göbel et al. 2007; Shelver et al. 2008). In this study, the high removal efficiencies of both sulfamethoxazole and NACS were observed under tropical climate conditions and no concentrations of sulfamethoxazole were detected in the effluent samples.

The other antibiotic (ciprofloxacin) was also completely removed (>99 %) in both HWWTPs. Gao et al. (2012) reported a lower removal efficiency (67 %) in a Chinese AS process. Ciprofloxacin is a fluoroquinolone and it is known that adsorption to sludge is a major removal process. For example, a significant amount of ciprofloxacin (up to 90 %) was removed via adsorption when the pH was less than 5.5 in a laboratory experiment (Githinji et al. 2011). However, adsorption has been observed to decrease with increasing temperatures (Seedher and Sidhu 2007) and an increase in pH (pH > 6) (Githinji et al. 2011). In this study, the removal efficiency of ciprofloxacin occurred under a high ambient temperature (>26 °C) and normal pH (7–8) conditions; this indicates that its removal was probably more as a result of biodegradation than adsorption.

#### Carbamazepine

The removal efficiency of carbamazepine by WWTPs has previously been found to be poor, mostly below 10 % (Zhang et al. 2008). Removal rates from literature suggest consistently low removal rates across treatment types as shown in Table 1. In fact, increases in carbamazepine concentrations after wastewater treatment have been reported (Joss et al. 2005; Vieno et al. 2007). In the HWWTPs in Saudi Arabia in this study, a high average removal efficiency of carbamazepine (>86 %) was observed. The HWWTP2 achieved >99 % removal of the compound. In Portugal, Dordio et al. (2010) reported similar removal efficiencies in wastewater collected in summer conditions (97 % removal, average temperature 26 °C), but lower levels in wastewater collected in winter conditions (88 % removal, average temperature 12 °C), indicating an effect of temperature on biodegradation rates. In addition to temperature effects, solar degradation could play a major role in the removal of carbamazepine. Donner et al. (2013) found that after 120 min of UV treatment, the concentrations of carbamazepine in solution were decreased to approximately 1 % of the starting concentration. High ambient temperatures and exposure to sunlight in Saudi Arabia may also have played a role in the high removal efficiencies observed for carbamazepine. These findings were unexpected; they suggest that conventional WWTPs could remove carbamazepine under certain conditions and that tropical climate conditions are potentially more favorable than temperate ones.

#### Atenolol

The average removal efficiency of atenolol by the HWWTPs was 89 %. The removal efficiencies of this compound reported in the literature vary drastically from study to study. For example, in WWTPs located in temperate climates (in Europe), Paxeus (2004) reported a removal efficiency of <10 %, while Vieno et al. (2005) reported a removal efficiency of 61 %. Castiglioni et al. (2006) found that the removal efficiency of atenolol was affected by temperature, where higher removal efficiencies were achieved in summer (55 %) than in winter (10 %). This indicates that the high removal efficiencies achieved by the HWWTPs in Saudi Arabia observed in this study could be due to higher microbial activity in the tropical climate.

#### Lidocaine

The average removal efficiency of lidocaine by the HWWTPs was 64 %; however, the difference between removal at HWWTP1 and HWWTP2 was large with 28 and >99 % removal observed respectively. The removal efficiency of lidocaine was assessed at various WWTPs in a temperate climate (Hesse, Germany), where it was found to be significantly lower (10–50 %) (Rúa-Gómez and Püttmann 2012) than that observed in this study. Kovalova et al. (2012) also found only moderate removal of lidocaine in a pilot-scale MBR treating hospital wastewater.

## Discussion of the importance of key factors on removal efficiency

The removal efficiencies of selected pharmaceutical compounds measured in this study appeared to be much improved in the hotter, tropical climate of Saudi Arabia.

Other studies have found that higher removal efficiencies are observed during the summer in temperate climates, by an average of 25 %, compared to winter (Vieno et al. 2005). In another study, which examined six large WWTPs in Italy, Castiglioni et al. (2006) also found higher removal efficiencies in summer (18.6 °C) than in winter (9.7 °C), except for the removal efficiencies of carbamazepine and ciprofloxacin, which were similar across the two seasons. In this study, >99 % removal efficiencies in relation to ciprofloxacin at both HWWTP and carbamazepine at HWWTP2 were achieved. In addition, very high removal efficiencies regarding the antibiotics, atenolol, and lidocaine were achieved; these compounds are normally found to be persistent in conventional WWTPs in temperate climates (Paxeus 2004; Rúa-Gómez and Püttmann 2012; Carballa et al. 2004). Thus, the higher ambient temperatures (>26 °C), which are present almost year-round in the tropical Saudi Arabian climate, may have enhanced the removal efficiencies of these compounds. This is because the tropical conditions may have led to a high level of microbial activity during the AS process, which may, in turn, have increased the biodegradation kinetics. This theory is supported by the knowledge that microorganisms living in reactors at WWTPs usually reach their optimal activity rates at warm temperatures, 25–35 °C (Cruikshank and Gilles 2007; Kareem 2013).

Other factors, such as sunlight availability (which is important for photodegradation), may also have influenced the removal efficiency of the pharmaceutical compounds (Kasprzyk-Hordern et al. 2009), particularly carbamazepine (Donner et al. 2013). It should be noted, however, that removal from effluent does not necessarily result in reduced toxicity. In the experiments by Donner et al. (2013), UV exposure was found to coincide with both a decrease in carbamazepine, but also an initial increase in degradation products acridine and acridone, which were shown to be significantly more toxic in acute toxicity assays than carbamazepine. More comprehensive studies are needed to investigate the multiple factors that cause parent compound degradation, but potential formation of recalcitrant degradation byproducts, and the relative toxicity.

In addition, the HWWTPs assessed in this study applied tertiary treatments, in the forms of sand filtration and disinfection. It is possible that sand filtration had an effect on the removal efficiencies of the pharmaceutical compounds. However, the removal of pharmaceutical compounds during sand filtration has generally been reported to be inefficient (Hollender et al. 2009; Nakada et al. 2007; Lin et al. 2010; Kosma et al. 2010). Therefore, in this study, although the samples were collected after the final treatment, in the interpretation of the results, it has been assumed that sand filtration played negligible roles in the fate of the target micropollutants in the plants. However, the contribution of the tertiary treatments to the removal of the pharmaceutical compounds requires investigation.

The role of chlorination may also be significant for some compounds. Removal of antibiotics, including sulfamethoxazole and ciprofloxacin (Li and Zhang 2012; Dodd et al. 2005), endocrine disrupting compounds and anti-inflammatory drugs (Noutsopoulos et al. 2015) have been shown to be affected by chlorination, with pH influencing the level of removal achieved. For acidic pharmaceuticals, some compounds have been found to degrade significantly due to chlorination (salicylic acid, naproxen, diclofenac, indomethacin); however, chlorine does not seem to lead to degradation of others (bezafibrate, ketoprofen, ibuprofen) (Quintana et al. 2010).

Finally, only the liquid wastewater was tested in this study and it is possible that the pollutants were adsorbed onto the sludge. It is noted that some substances will be more prevalent to sorption to sludges than others. Carbamazepine for example displays extremely low sorption to sludges, and sulfamethoxazole shows low sorption therefore removal by sorption to sludge is unlikely to be a primary removal mechanism for these compounds, whereas atenolol displays moderate sorption therefore some removal may be due to sorption (Horsing et al. 2011). This is an area for further study and investigation in tropical climates.

## Conclusions

The onsite HWWTPs in Saudi Arabia achieved high removal efficiencies from wastewater of the pharmaceutical compounds tested, including ciprofloxacin, clarithromycin, sulfamethoxazole and NACS, paracetamol, caffeine, atenolol, lidocaine, and carbamazepine, from the wastewater. The high removal efficiencies of carbamazepine, in particular, were unexpected due to the recalcitrant nature of this compound. Temperature, and potentially photodegradation, were identified to be factors that probably led to the high removal efficiencies achieved. More work is needed to confirm the role that these, and other potential factors, play.
